# Cancer Survivorship Care: Person Centered Care in a Multidisciplinary Shared Care Model

**DOI:** 10.5334/ijic.3046

**Published:** 2018-01-16

**Authors:** Jacqueline J Loonen, Nicole MA Blijlevens, Judith Prins, Desiree JS Dona, Jaap Den Hartogh, Theo Senden, Eline van Dulmen-Den Broeder, Koos van der Velden, Rosella PMG Hermens

**Affiliations:** 1 Department of Hematology, Radboudumc, Geert Grooteplein-Zuid 8 (route 476), 6525 GA Nijmegen, NL; 2 Department of Medical Psychology, Radboudumc, Geert Grooteplein- zuid 10 (route 840), 6525 GA Nijmegen, NL; 3 Department Occupational Health, Radboudumc, Erasmuslaan 17 (route 980), 6525 GE Nijmegen, NL; 4 Dutch Childhood Cancer Parent Organization VOKK Netherlands, Department VOX Survivors, Schouwstede 2-B, 3431 JB Nieuwegein, NL; 5 Department Academic Workplace Care for Work, Radboudumc, Geert Grooteplein-zuid 10 (route 623), 6525 GA Nijmegen, NL; 6 Department of Pediatrics, Division Pediatric Oncology/Hematology, VU University Medical Center, De Boelelaan 1117, 1081 HV Amsterdam, NL; 7 Department of Primary and Community Care, Radboudumc Geert Grooteplein-noord 21, 6525 EZ Nijmegen, NL; 8 Radboud Institute for Health Sciences (RIHS), Scientific Institute for Quality of healthcare (IQ healthcare), Radboudumc, Geert Grooteplein-noord 21 (route 114), 6525 EZ Nijmegen, NL

**Keywords:** Cancer survivorship care, Person centered care

## Abstract

Survivors of childhood and adult-onset cancer are at lifelong risk for the development of late effects of treatment that can lead to serious morbidity and premature mortality. Regular long-term follow-up aiming for prevention, early detection and intervention of late effects can preserve or improve health. The heterogeneous and often serious character of late effects emphasizes the need for specialized cancer survivorship care clinics. Multidisciplinary cancer survivorship care requires a coordinated and well integrated health care environment for risk based screening and intervention. In addition survivors engagement and adherence to the recommendations are also important elements. We developed an innovative model for integrated care for cancer survivors, the “Personalized Cancer Survivorship Care Model”, that is being used in our clinic. This model comprises 1. Personalized follow-up care according to the principles of Person Centered Care, aiming to empower survivors and to support self management, and 2. Organization according to a multidisciplinary and risk based approach. The concept of person centered care is based on three components: initiating, integrating and safeguarding the partnership with the patient. This model has been developed as a universal model of care that will work for all cancer survivors in different health care systems. It could be used for studies to improve self efficacy and the cost-effectiveness of cancer survivorship care.

## Introduction

The population of cancer survivors worldwide is rapidly increasing because of greatly improved survival rates in both childhood and adult-onset cancers [1]. The down side is that successful cancer treatment predisposes survivors to an elevated life-long risk of treatment related adverse health effects [2,3]. These late effects can be serious, leading to chronic morbidity and premature mortality, and may not become apparent before years or even decades after finishing treatment [3]. The life-long increased risk of chronic health conditions in the increasing cancer survivor population underscores the importance to address the health concerns of cancer survivors. The need for regular, long-term follow-up care for effective prevention or intervention to preserve or to improve health of cancer survivors has been recognized [4]. Models to deliver cancer survivorship care are evolving [5]. Achieving high-quality cancer survivorship care requires, besides evidence based guidelines for screening and surveillance, a comprehensive, multidisciplinary care infrastructure, coordination between specialists and primary care providers, a survivor care plan with a summary of treatment and a model that enables care assessment with the overarching goal of improving quality of care [4,6]. As late effects can occur decades after treatment, from the survivors’ perspective, optimal engagement in their own follow-up care is an important aspect. Therefore cancer survivorship care should also include personalization of care, aiming to empower survivors and to support self management. In addition, considering limitation on health care resources, provision of follow-up care needs to be sustainable and cost effective [7]. In a recent issue of the Lancet Oncology the need to identify effective and efficient models of care has been emphasized [8]. The different aspects of cancer survivorship care require innovative solutions in a cost effective way. We developed an integrated, shared care model of personalized follow-up care, the so called Personalized Cancer Survivorship Care Model, based on a multidisciplinary and risk based approach for childhood and adult-onset cancer survivors. The model, based on the principles of Person Centered Care as developed by Eckman et al and the guidelines of the American Association of Clinical Oncology, has been used in our University Medical Center for more than one year and has shown to be a universal model of care for all cancer survivors [4,9]. It can be used for studies to the self-efficacy and the cost-effectiveness of cancer survivorship care.

## Person Centered care

Transition from cancer patient to cancer survivor requires a change in approach of both health care provider and survivor. The emphasis changes from protocol driven cancer treatment and disease surveillance, to personalized care that meets all the physical, mental and social health needs of the survivor, thereby respecting survivors’ preferences and health values. Also the role of patients in the healthcare system is changing. Patients are no longer passive receptors of medical prescription but move towards partnership taking an active role in managing their health [10]. A concept that actively involves patients as partners is the concept of Person Centered Care [9]. Person centered care has been shown to advance harmonization between care provider and patient on treatment plans, to improve health outcomes and to increase patient satisfaction [10,11,12]. Therefore person centered care has been advocated to be a key component of high quality care [9,10,11,12]. Person centered care is based on three components: initiating, integrating and safeguarding the partnership with the patient [9]. Important for the initiation of partnership is to set the person’s perspective on his or her life situation and health condition, at the center of care. Integration of partnership requires sharing information, with respect to the persons’ narrative and understanding of the persons values and preferences regarding their care. The registration of information on the persons’ preferences and needs regarding their health and health concerns is crucial for safeguarding the partnership [9].

Principle requirements for person centered care align with important elements in cancer survivorship care. Initiating the partnership, by putting survivors’ narrative at the center of care and considering his or her needs, values and preferences, aligns with the risk-stratified approach to care. This approach is based on the medical history and treatment summary which is important in cancer survivorship care. Integration of partnership needs a well informed patient, a coordinated care environment, and harmonization and sharing of care between the caregivers, which requires the coordination of multidisciplinary care, again an important element in cancer survivorship care. Safeguarding the partnership aligns with the development of a survivor care plan [6]. So aiming to empower and encourage survivors to take an active role in managing their health, person centered care has been implemented in the Personalized Cancer Survivorship Care model.

## The Personalized Cancer Survivorship Care model

The Personalized Cancer Survivorship Care Model is based on two major principles: 1 Successful implementation of person centered care which depends on three factors: an informed and involved patient, receptive and responsible health professionals and a coordinated and well-integrated health care environment [12], and 2 clinical practice guidelines for screening and surveillance on late effects. Clinical practice guidelines are extremely important to facilitate early detection and treatment of (a)symptomatic late effects. As such clinical practice guidelines are considered as important tools to improve quality of care and to reduce costs [13]. Therefore guidelines are basic elements in the personalized cancer survivorship care model. Guidelines addressing the surveillance for late effects in long-term survivors of childhood and young adult cancer have been published by the North American Children’s Oncology Group, the Dutch Childhood Oncology Group, the United Kingdom Children’s Cancer and Leukaemia Group, the Scottish Intercollegiate Guidelines Network and the guidelines of the International Guideline Harmonization Group [13,14,15,16,17,18]. Guidelines for older survivors of adult onset cancer are available as well [19,20,21].

**Figure 1:**
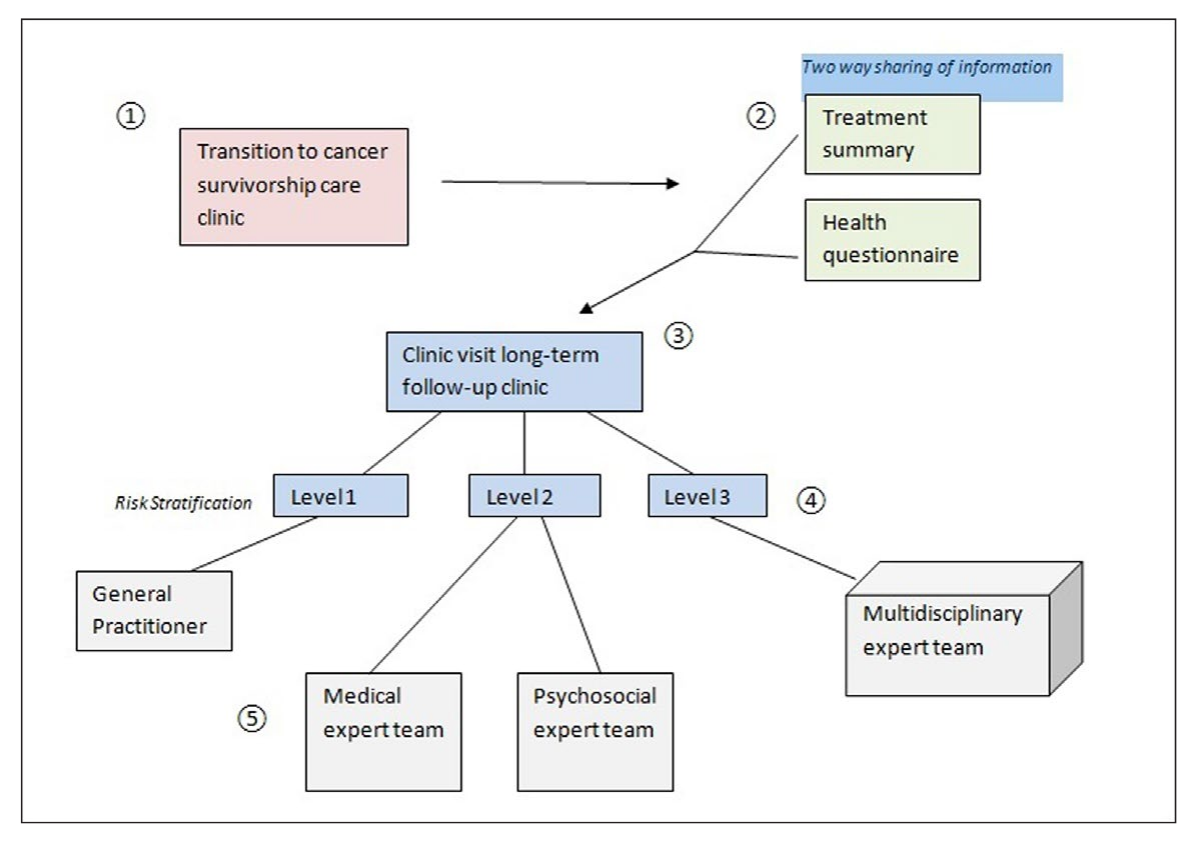
**Model of Personalized Cancer Survivorship Care.** Pathway of cancer survivors through the care pathway. 1. Transition to the survivor care clinic; 2. Two-way sharing of information; 3. Clinic visit for screening, health promotion and disease prevention; 4. Risk Stratification; 5. Shared Care.

Besides these two major principles, the following 5 steps are important for PCSC (Figure 1).

## The development of specialized Cancer Survivorship Care Clinics

Many hospitals in Europe, the USA, Canada and Australia set up survivorship clinics for risk-based longitudinal follow-up of childhood cancer survivors. The current development is to extend cancer survivorship care also to survivors of adult-onset cancer with a high risk for late effects after treatment. The Dutch Childhood Oncology Group long term follow-up guidelines recommend transition of cancer survivors to the survivorship clinic when they are five years from diagnosis and in remission of their malignant disease.

## Two way sharing of information/Risk based screening

Initiating the partnership according to the concept of person centered care starts with a two-way sharing of information before the clinic visit. The health care provider creates a summary of the medical history including a treatment summary and survivors will be asked to fill in a questionnaire on health related problems. As frequency and severity of late effects depend on the type of malignancy and the treatment that survivors received, a treatment summary is mandatory for a risk-based screening on treatment-related late effects [6]. Still the clinical requirements of cancer survivors will vary from person to person as the risk and manifestation of late effects in an individual survivor is also influenced by other factors including premorbid health conditions, genetic or familial characteristics, and life style factors [5,6]. Also adverse psychosocial effects of cancer on educational achievement or employment status may affect the course of late effects. Therefore survivors have to be requested to fill in a questionnaire regarding their health problems, health behavior and medical and psychosocial needs. Sharing information in preparation to the clinic visit facilitates effective communication during the consultation, and enables to put the survivors narrative at the center of care.

## Clinic visit

The long-term follow-up program provides, besides screening for late effects, information on health promotion and disease prevention [22]. Health issues to be addressed during the clinic visit are: Physical health and function, mental health, sexual and reproductive health, social outcome, health behavior and health education [22]. To ensure integration of the partnership, decisions regarding intervention and prevention are made considering survivors’ needs, values and preferences, using shared decision making.

## Risk-stratification

Not all survivors will need life-long follow up care in a specialized survivor clinic. To provide care appropriate to the needs, preferences and health values of the survivor and to avoid unnecessary care, Wallace et all developed a three level model to guide decisions about intensity and frequency of follow-up care [23,24]. This risk-stratified approach, also according to the DCOG guidelines, has been incorporated in the model for personalized cancer survivorship care.

Based on these levels three different pathways for follow-up care are designed. Each survivor is stratified to a pathway depending on the risks for late effects, specifically: level 1 care, for survivors without elevated risk for the development of late effects, can be provided by a Primary Care Physician, survivors of level 2 need lifelong follow-up care in a cancer survivorship care clinic because of the risks on late effects later in life and survivors of level 3 require complex care by a multidisciplinary team in the survivorship care clinic. Apart from the risk-stratified approach according to the three level model, on the individual level, the anticipated health care needs of the survivors may differ markedly. Therefore referral of all cancer survivors to the survivor clinic for screening, surveillance and prevention would be advised.

## Multidisciplinary Shared Care

Multidisciplinary shared care according to the personalized cancer survivorship care model requires both vertical and horizontal integration of care. Vertical integration of care is collaboration through the healthcare system: General Physician to local hospital to University Medical Center. Horizontal Integration of care is collaboration within the expert teams of the University Medical Center. The physician or specialized nurse in the survivor clinic is responsible for the coordination of both the vertical and horizontal integration of care. The pathway for follow-up after the clinic visit is tailored to late effects, comorbid conditions and needs of the survivor. The primary care physician is involved in the provision of follow-up care for survivors who have been treated with low risk chemotherapy or surgery alone (level 1). These survivors have a minimal risk for late-occurring health problems related to cancer therapy and do not need follow-up in a specialized center. Apart from follow-up, primary care physicians are also involved in intervention for first line treatment e.g. hypertension and hypothyroidism.

To facilitate comprehensive care for survivors at risk for or with serious late effects (level 2 and 3) and survivors at high risk for multiple health problems e.g. survivors of brain tumors (level 3), the survivorship care team has to be extended with a team of dedicated specialists with interest in cancer survivorship care (Table 1).

**Table 1:** Healthcare team for specialized cancer survivorship care.

Survivorship clinic/coordination	(Pediatric) Oncology Physicians, Specialized nurses
Psychosocial Expert team:	Neuropsychology with expertise in neurocognitive function, Psychology with expertise in the treatment of fatigue, Social Worker, Occupational Health Physician
Medical Expert team:	Cardiology, Endocrinology, Neurology, Gynecology and urology with expertise in reproductive health and fertility issues, Nephrology, Dermatology, Rehabilitative services, Chest Physician, Specialized nurse breast cancer surveillance
Consultants:	Clinical Geneticist, Dentist, Dietician

The cooperation and horizontal integration of a multidisciplinary expert team facilitates access to appropriate care for survivors with different late effects. The specialists of the expert-team cooperate with specialists in the local hospitals for survivors with serious late effects that will need life-long follow-up care. Irrespective of referral to the primary care physician or specialist, the pathway for survivors graded to level 2 and 3, comprises lifelong routine follow-up in the survivorship clinic for screening and surveillance of late effects, every 1 (< the age of 18 years); 2, 5 or 5 years, according to the guidelines [14,15,16,17,18,19,20,21].

## Organization of Personalized Cancer Survivorship Care on an individual level

**Figure 2:**
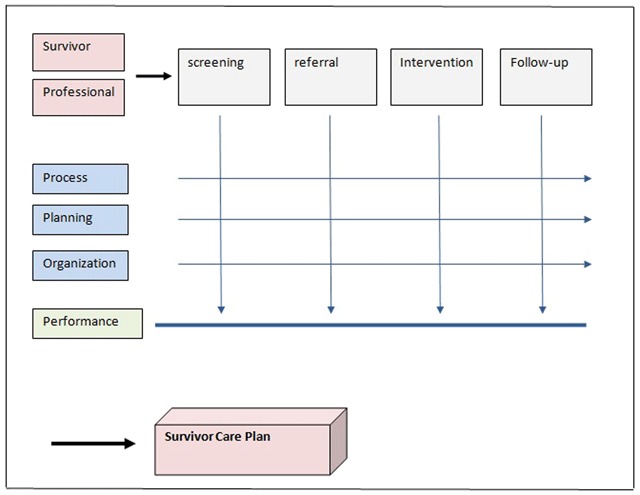
**Integrated Oncology Care Pathway model.** The infrastructure of the Comprehensive Cancer Center The Netherlands (IKNL) model of Integrated Oncological Care Pathways. Input of survivors and professionals on the care process. Description of process, planning and organization. Assessment of performance. Summary in a survivor care plan.

In addition to the 5 steps model of the personalized cancer survivorship care it is also important to organize cancer survivorship care on an individual level. Providing high quality person centered care to survivors facing different late effects, leading to pathways to specialists of a multidisciplinary team, requires an infrastructure in which comprehensive, integrated care, according to the guidelines, can be delivered. To achieve all these objectives on an individual level, the infrastructure of the Comprehensive Cancer Center The Netherlands model of Integrated Oncological Care Pathways was implemented in the model of personalized cancer survivorship care [25,26,27] (Figure 2).

### The model of Integrated Oncological Care Pathways: (Figure 2)

The Integrated oncology care pathway model is based on principles of process management and the use of guidelines [25]. A major advance of the model is that input of professionals e.g. adjustment of guidelines, and input of survivors on their preferences how to organize the long-term follow-up care, can easily be implemented. Central elements in this model are agreement of both professionals and survivors, on the process, organization and planning. In cancer survivorship care, each survivor will have his or her own pathway depending on survivors ‘needs, the occurrence of late effects and co-morbid conditions. In the oncology care pathway model the process management of the different aspects of care: screening, referral to the primary care physician or one of the specialists of the expert team, intervention and follow-up will be organized in a uniform way with clear identification of roles and responsibilities. The oncology care pathway model is a solid basis for a coordinated and well-integrated health care environment, that may improve the performance of the care pathway and results in a better quality of care [26]. In addition performance of the different aspects of care can be assessed. Preliminary evaluation shows that survivors report very positive experiences with this integrated care.

### Survivor care plan

An integral component of cancer survivorship care is a survivor care plan, which is regarded as a core measure of the American Society of Clinical Oncology Quality Oncology Practice Initiative [4,28]. After the clinic visit survivors will be provided with a personalized survivorship care plan that contains a treatment summary, possible long-term effects and interventions, guidelines for follow-up care and lifestyle recommendations.

The survivor care plan is an essential basis for both safeguarding the partnership and facilitating the integration of shared care as it can clearly delineate which provider is responsible for which aspect of care. It is important to engage survivors in the survivorship care planning process as it will promote shared decision making, self management and patient engagement, which are associated with adherence to recommendations [28].

In countries as the Netherlands and Australia, many survivors have full access to their data in their own electronic health record and to improve utility, the survivor care plan will be integrated in survivors’ electronic health records. The use of the survivor care plan will facilitate communication between the physician in the survivor clinic, the survivor and healthcare providers through the healthcare system.

### Cost effectiveness

New models of care will have an economic impact and cancer survivorship care of the increasing cancer survivor population requires a cost-effective solution. Although the cost-effectiveness of the proposed personalized survivorship care model needs to be assessed, the different elements of the cancer survivorship care: Person centered care [9], care delivery according to guidelines [13], and care according to oncology care pathways [26] have been demonstrated to be cost-effective. In a recent study Sutradhar et al demonstrated that attendance at a specialized survivor clinic resulted in fewer visits to the Emergency Department [29] and two studies showed that cardiac care assessment according to the guidelines of the Children’s Oncology Group may reduce cardiac failure incidence in a cost-effective way [30,31]. Armstrong et al reported in a large cohort study a reduction in late mortality among 5-year survivors of childhood cancer in more recent eras [32]. Although this improvement also reflects the reduction of treatment exposures during cancer treatment in order to minimize long-term effects, a potential contributor can be the use of screening and early intervention in the late effects clinics. The next step is to further determine whether specialized survivorship clinics reduce hospitalization rates and lead to a further reduction of both serious morbidity and premature mortality in long-term cancer survivors in a cost effective way. In future research we want to investigate the self efficacy and the cost effectiveness of the Personalized Cancer Survivorship Care Model.

## Conclusion

The presented model of personalized Cancer Survivorship Care demonstrates a structure of comprehensive cancer survivorship care through a specialized survivorship clinic providing and coordinating care for cancer survivors that is complemented, through an integrated shared care model, with care of the primary care physician and local hospital care. This model turned out to be a universal model of care that works for all cancer survivors. This model can be used for studies to the self efficacy and the cost-effectiveness of cancer survivorship care.
